# PRAK mediates Aβ-RAGE driven autophagy pathway

**DOI:** 10.18632/oncotarget.14544

**Published:** 2017-01-06

**Authors:** Yoonhee Kim, Inhee Mook-Jung

**Affiliations:** ^1^ Department of Biochemistry and Biomedical Sciences, Seoul National University College of Medicine, Seoul, Korea

**Keywords:** PRAK, RAGE, Aβ, autophagy, Alzheimer's disease

Alzheimer’s disease (AD) is an irreversible progressive neurodegenerative disorder, resulting in loss of memory and behavioral changes. It is characterized by amyloid-β (Aβ) plaques, neurofibrillary tangles, and synaptic loss. Aβ affects AD pathogenesis, including oxidative stress, inflammatory response, and neuronal death. The intermediate mechanism between Aβ toxicity and AD pathogenesis is a subject of ongoing investigation. So far, there is no disease-modifying drug for AD and the available FDA-approved drugs are only useful for the symptomatic treatment of the patients [1].

The receptor for advanced glycation end products (RAGE), which has multi-ligands including AGE, S100, and Aβ, has recently attracted much attention for its involvement in various diseases such as type 2 diabetes, cardiovascular disease, and AD. Although RAGE-mediated cellular signaling is reported in inflammation, apoptosis, cell migration, and autophagy [2], the lack of a signal transduction motif in the cytoplasmic domain of RAGE raises a question regarding the mechanism by which downstream signals are triggered inside the cell.

We have recently shown that Aβ-RAGE interaction recruits p38-regulated/activated protein kinase (PRAK) to the RAGE cytoplasmic domain *in vitro* and *in vivo*. This direct binding between RAGE and PRAK has been confirmed by various biochemical tools such as yeast two hybrid (Y2H) screening, immunoprecipitation (IP), and surface plasmon resonance (SPR) assay [3].

PRAK is a member of the MAPKs and can be activated by phosphorylation in response to cellular stress and proinflammatory cytokines. It plays a critical role in cellular homeostasis such as angiogenesis, cell cycle, and neuronal plasticity. Moreover, PRAK regulates the phosphorylation of Ras homolog enriched in brain (Rheb), a key component of mammalian target of rapamycin complex 1 (mTORC1) [4].

When Aβ binds to the RAGE, PRAK is activated through its interaction with the RAGE cytoplasmic domain. Experimental evidence showed that unlike the full-length human RAGE overexpressing cells, PRAK is not phosphorylated by Aβ treatment in the cells expressing the cytoplasmic domain deletion mutant of RAGE (DN-RAGE), suggesting that the cytoplasmic domain of RAGE is essential for the recruitment of PRAK upon Aβ binding to RAGE.

To investigate the effect of Aβ on PRAK-RAGE-mediated signaling pathway, we focused on the alteration of Rheb or mTORC1/p70S6k because PRAK is a kinase for Rheb in a cellular growth system [4]. Cells treated with Aβ showed increased levels of phosphorylated Rheb and p70S6k; however, these levels were not altered in the DN-RAGE-expressing or PRAK-silenced (siPRAK) cells. These data indicate that Rheb-mTORC1/p70S6k pathway is activated under Aβ stimulation and that it depends on the PRAK-RAGE interaction.

Several lines of evidence suggest that autophagy is involved in AD pathology and this concept can be utilized for the development of new therapeutic approaches for AD [5]. Since one of the signaling pathways in autophagy is the Rheb-mTOR pathway and Aβ-RAGE-PRAK axis activates the Rheb-mTOR signaling pathway, we further explored whether PRAK regulates autophagy via Aβ- RAGE interaction. To evaluate this possibility, we tested the phosphorylation levels of mTOR and UNC-51-like kinase 1 (ULK1), a marker for autophagy induction. After treatment of the cells with Aβ, the phosphorylation of mTOR and ULK1 was significantly decreased. In contrast, Aβ treatment did not alter the phosphorylation of mTOR and ULK1 in PRAK-silenced cells. In addition, knockdown experiments for PRAK inhibition blocked Aβ-induced autophagosome formation (not autophagic flux), implicating the crucial involvement of PRAK in Aβ- RAGE-induced autophagy induction.

Multiple evidences suggest that autophagy plays a beneficial role to remove Aβ accumulation. However, some of the previous studies have shown that the ability of autophagic clearance is defective in AD. The combination of induction of excessive autophagy and impaired clearance of autophagic vacuoles creates conditions favorable for Aβ deposition, as evidenced by extensive accumulation of autophagic vacuoles and massive buildup of incompletely digested substrates in the brains of patients with AD [5]. Since the accurate state and competence of lysosomes in each stage of the disease remain largely unknown, the role of autophagy in AD pathogenesis is still controversial [6]. Therefore, delineating the detailed mechanism of autophagic flux in AD might exploit the potential of autophagy modulation as a therapeutic strategy.

**Figure 1:**
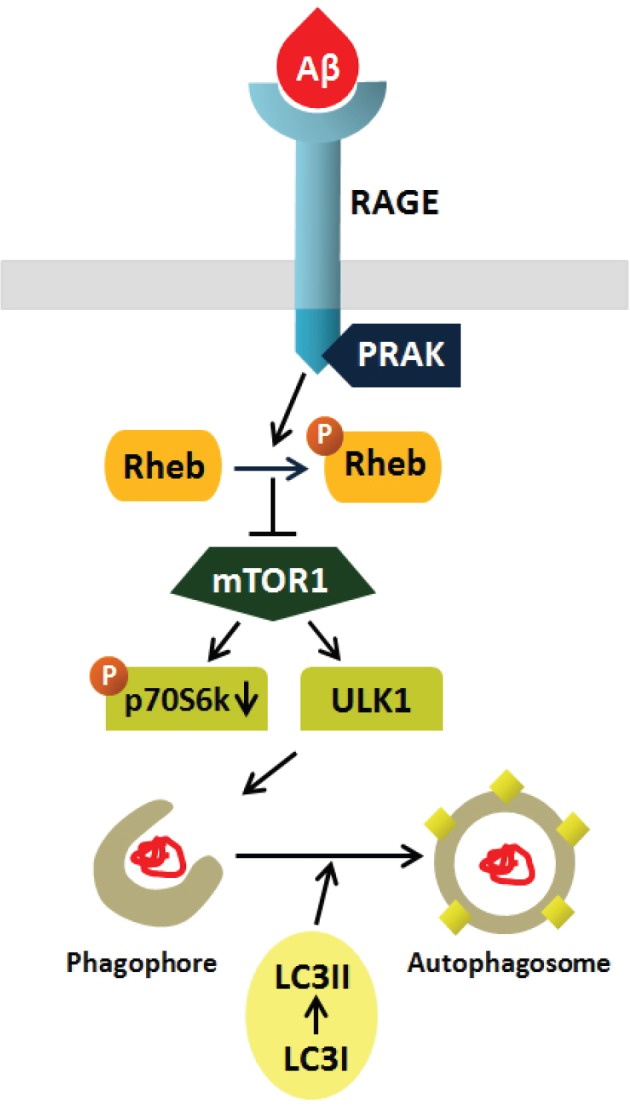
Schematic diagram of signal transduction from Aβ, RAGE and PRAK Aβ binds to RAGE which recruits PRAK to the RAGE cytoplasmic domain, leading to mTOR/ULK1 mediated autophagosome formation.

A key finding of our study is that PRAK might be a critical regulating factor of AD involved in RAGE-mediated autophagy induced by Aβ (Figure 1). Restoration of functional lysosomes and maintenance of efficient autophagic flux in AD pathology have yielded promising therapeutic results. Therefore, a better understanding of the involvement of PRAK and RAGE in autophagy will hopefully provide a new therapeutic strategy for AD. Besides autophagy, ligand-RAGE interactions can mediate diverse cellular signaling pathways that influence cellular homeostasis and inflammatory response leading to various diseases such as cancer, diabetes, vascular diseases, and neurodegenerative diseases [7]. Therefore, extensive work is needed to elucidate other signal transduction mechanisms and the involved molecules following ligand- RAGE interaction that might be potential therapeutic drug targets.
